# Alcohol-Associated Liver Disease Mortality Rates by Race Before and During the COVID-19 Pandemic in the US

**DOI:** 10.1001/jamahealthforum.2023.0527

**Published:** 2023-04-21

**Authors:** Neeti S. Kulkarni, Divneet K. Wadhwa, Fasiha Kanwal, Jagpreet Chhatwal

**Affiliations:** 1 Institute for Technology Assessment, Massachusetts General Hospital, Boston; 2 Harvard Medical School, Boston, Massachusetts; 3 Department of Gastroenterology, Baylor College of Medicine, Houston, Texas

## Abstract

This cross-sectional study examines the national- and state-level age-adjusted mortality rates for alcohol-associated liver disease in 4 racial groups, with a focus on the American Indian or Alaska Native population.

## Introduction

In 2021, US life expectancy dropped to its lowest level since 1996, with the largest decline among the non-Hispanic American Indian or Alaska Native population.^1^ The leading cause of death for this group, other than COVID-19 and unintentional injuries, was chronic liver disease (CLD).^1^ Recent growth in CLD prevalence is primarily associated with an increase in alcohol-associated liver disease (ALD).^2^ Rates of ALD increased by 43% from 2009 to 2015, accounting for over $5 billion in direct health care costs in 2015.^3^ This problem was exacerbated by the COVID-19 pandemic, during which ALD deaths increased by 22.4%.^4^ Given the acceleration of ALD and the burden of CLD for American Indian or Alaska Native populations, we conducted a study to understand the association between race and ALD mortality at the state level before and during the pandemic.

## Methods

We obtained 2019 and 2020 data from the Centers for Disease Control and Prevention (CDC) WONDER Multiple Cause of Death database. In accordance with the Common Rule, the study was exempt from review and the informed consent requirement because it used publicly available data and was not human participant research. We followed the STROBE reporting guideline.

Age-adjusted mortality rates (AAMRs) per 100 000 people were extracted for alcohol-associated CLD (*International Statistical Classification of Diseases and Related Health Problems, Tenth Revision* code K70) by state and race: American Indian or Alaska Native, Asian or Pacific Islander, Black or African American, and White populations, as classified in CDC WONDER.

## Results

**Figure 1.**
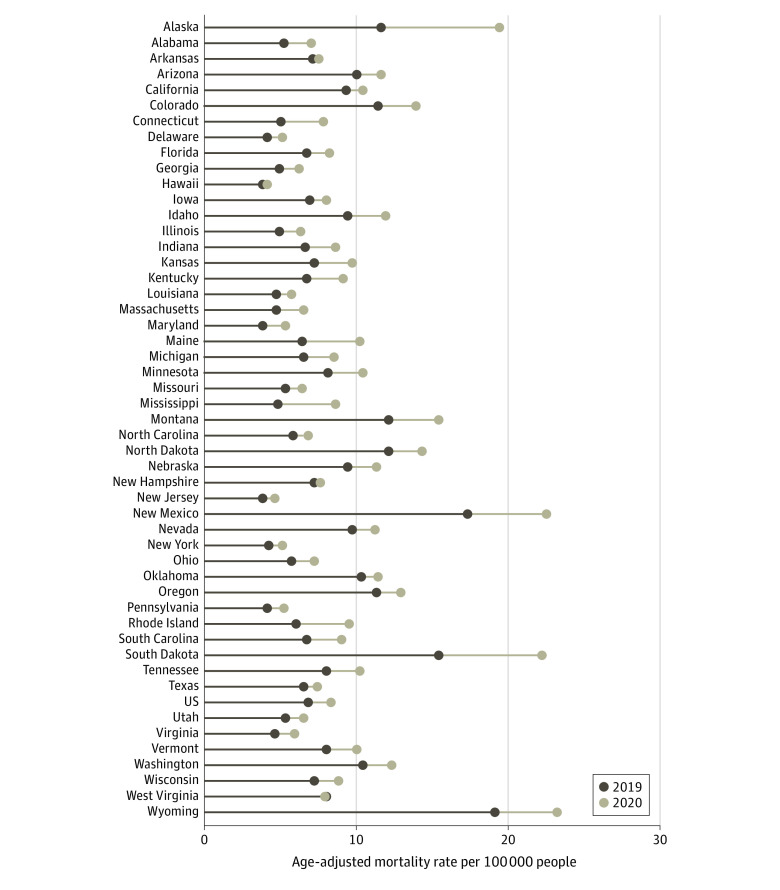
Age-Adjusted Mortality Rates for Alcohol-Associated Liver Disease by State for 2019 and 2020

**Figure 2.**
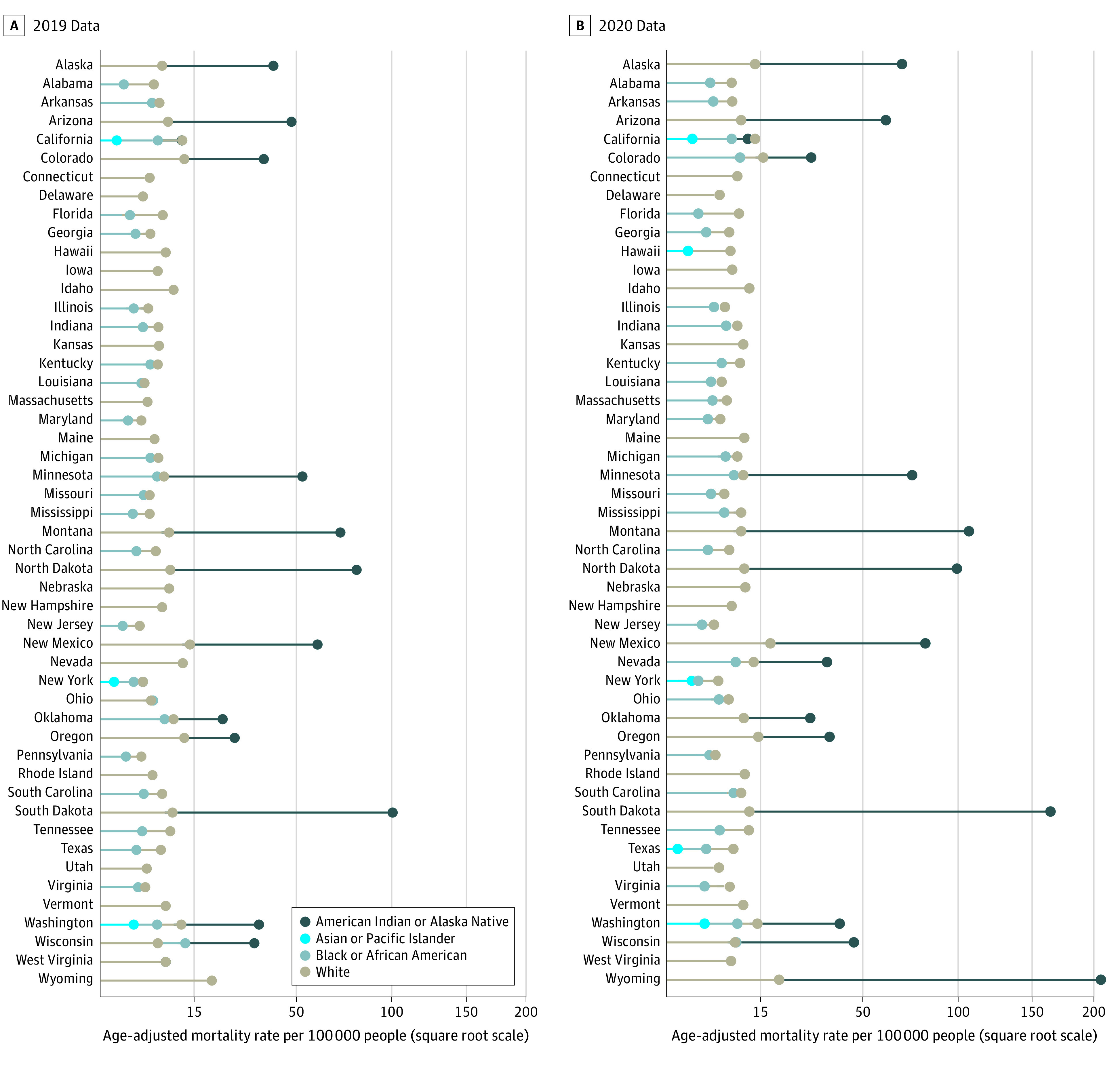
Age-Adjusted Mortality Rates for Alcohol-Associated Liver Disease by State for 2019 and 2020, Stratified by Race A square root scale was used on the x-axis given the differences in rates between the racial and ethnic groups. Data were suppressed when death counts were less than 10.

From 2019 to 2020, the national AAMR for ALD increased by 23.4%, from 6.4 to 7.9 per 100 000 people, whereas state-level AAMRs increased in 49 of 50 states (Figure 1). The AAMRs increased for all groups from 2019 to 2020, with the largest national increase of 34.3% (from 20.1 to 27.0 per 100 000 people) among American Indian or Alaska Native individuals (Figure 2).

The highest AAMR in 2019 was 95.3 per 100 000 people in South Dakota and in 2020 was 199.4 per 100 000 people in Wyoming, both among American Indian or Alaska Native people. Among states with available data, the mean AAMR in this population was nearly 6-fold higher than in White people, the group with the second highest rates (68.5 vs 11.7 per 100 000 people). In these states, American Indian or Alaska Native populations accounted for 9.6% of all ALD deaths, yet represented only 3.2% of the 2020 total population.

## Discussion

Prevalence of ALD increased during the COVID-19 pandemic in the US, particularly among American Indian or Alaska Native populations. States with the highest general ALD AAMRs corresponded to states with the highest ALD AAMRs for American Indian or Alaska Native people. Although alcohol consumption is less likely among this group than other racial and ethnic groups, those who do drink are more likely to engage in excessive drinking, a factor in ALD.^5^ However excessive drinking is not the only factor associated with ALD mortality. Systemic failures, such as insufficient preventive care and underfunded resources, also contribute to high mortality for American Indian or Alaska Native individuals.^5^

Study limitations include data suppression for low population counts and heterogeneity of American Indian or Alaska Native populations across the country. Furthermore, death certificates often classify race based on personal observations of funeral directors, which could lead to racial misclassification of American Indian or Alaska Native people.^6^

Currently, ALD represents a significant burden on the US health care system, and the problem could worsen over time. American Indian or Alaska Native individuals are particularly vulnerable to ALD; thus, policy actions must be taken, including wide implementation of universal alcohol screening and provision of federal and state resources for ALD through the Indian Health Services and Urban Indian Organizations in both tribal and urban areas. Such actions could help mitigate the growing ALD burden.

## Notes

Supplement.
Data Sharing Statement
