# Supplementary material for: Alcohol-Associated Liver Disease Mortality Rates by Race Before and During the COVID-19 Pandemic in the US
Source: JAMA Health Forum. 2023 Apr 21;4(4):e230527. doi: 10.1001/jamahealthforum.2023.0527 (PMC10122166; doi:10.1001/jamahealthforum.2023.0527)
Supplement: Supplement. — Data Sharing Statement [file jamahealthforum-e230527-s001.pdf]

Data Sharing Statement

Kulkarni. Alcohol-Associated Liver Disease Mortality Rates by Race Before and During the COVID-19 Pandemic in the US. *JAMA Health Forum*. Published April 21, 2023. doi:10.1001/jamahealthforum.2023.0527

Data
Data available: Yes
Data types: Other (please specify)
Additional Information: Publicly available data
How to access data: https://wonder.cdc.gov/controller/datarequest/D77
When available: With publication

Supporting Documents
Document types: None

Additional Information
Who can access the data: Everyone (publicly available data)
Types of analyses: Any analyses
Mechanisms of data availability: NA
